# HOME-BASED REMINISCENCE INTERVENTION IN DEMENTIA: QUALITATIVE THEMATIC ANALYSIS

**DOI:** 10.1093/geroni/igae098.3078

**Published:** 2024-12-31

**Authors:** Xianglan Jin, Kuan-Ching Wu, Shao-Yun Chien, Oleg Zaslavsky

**Affiliations:** University of Washington, Seattle, Washington, United States; University of Washington, Seattle, Washington, United States; University of Washington, Seattle, Washington, United States; University of Washington, Seattle, Washington, United States

## Abstract

Reminiscence intervention has shown promising potential in alleviating dementia-related morbidities, thanks to the relatively intact remote memories of persons living with dementia (PLWDs). However, reminiscence interventions have been primarily delivered in group settings by healthcare professionals, which is costly and hard to scale up. One innovative solution is training family caregivers to carry out reminiscence conversations with their loved ones in their homes at their convenience. This thematic analysis aims to examine the participants’ perspectives of a home-based reminiscence intervention to inform future research on dyadic reminiscence interventions. A pilot study on a home-based reminiscence intervention was conducted between November 2022 and May 2023. Qualitative data were collected through semi-structured individual interviews with nine dyad participants at the end of the intervention. The interviews were audio-recorded and transcribed verbatim. Initial codes and categories were developed inductively, and queries on Atlas.ti were used for theme identification and recontextualization. Peer debriefing and external review were utilized to ensure rigor. Participants reported positive experiences of the home-based intervention. Centering on and engaging the PLWD in conversations was perceived as positive brain stimulation. Participants highly appreciated that it was conducted in home settings and acknowledged that the intervention content, duration (1-1.5 hours for 24 weeks), frequency (biweekly), and logistics were acceptable. PLWDs expressed willingness to share stories with their families. Most caregivers showed great interest in training on reminiscence conversations. Future studies should empower family caregivers to initiate and lead reminiscence conversations at home along with the provision of adequate caregiving support.

